# Effectiveness of acupuncture in postpartum depression: A protocol for an overview of systematic reviews

**DOI:** 10.1097/MD.0000000000028678

**Published:** 2022-08-12

**Authors:** Bu Fan, Yonghou Zhao, Jianbo Chai, Bai Bing, Wanyu Wang

**Affiliations:** a Heilongjiang University of Chinese Medicine, Harbin, Heilongjiang Province, China; b Department of Neurology, Heilongjiang Mental Hospital, Harbin, Heilongjiang Province, China; c Postdoctoral Station of Integrated Medicine, Fudan University, Shanghai, China.

**Keywords:** acupuncture, assessment of multiple systematic reviews-2, GRADEE, overview, postpartum depression, preferred reporting items for systematic reviews and meta-analyses

## Abstract

**Introduction:**

Since conflicting evidence from systematic reviews and meta-analyses (SRs/MAs) on the effectiveness of acupuncture in the treatment of postpartum depression is observed. To systematically collate, appraise and synthesize the evidence from these SRs/MAs, an overview will be performed, and this study is an overview protocol.

**Methods and analysis:**

Eight databases will be searched: Medicine, Web of science, Cochrane Library, Embase, China National Knowledge Infrastructure, SinoMed, VIP, and Wanfang Data. SRs/MAs of acupuncture on postpartum depression will be included. Literature screening, data extraction, and evaluation of the review quality will be performed by 2 independent reviewers. The methodological quality, reporting quality, and evidence quality will be assessed using the assessment of multiple systematic reviews-2 tool, the preferred reporting items for systematic reviews and meta-analyses checklists, and the grading of recommendations, assessment, development, and evaluation system, respectively. The results will be presented in the context of the topic and the objects of the overview. This study will help bridge the implementation gap between clinical evidence and its translation in clinical application, identify flaws in research and guide future high-quality study.

## 1. Introduction

Postpartum depression (PPD) is a type of mood disorder associated with childbirth, the onset of which usually begins between the first day and 4months after delivery.^[1]^ Typical PPD occurs within 6 weeks postpartum and recovers on its own within 3 to 6 months, while severe cases can persist for 1 to 2 years. The prevalence of PPD in new mothers is as high as 16%,^[2]^ and the recurrence rate of PPD in the second pregnancy reaches 30%.^[3,4]^ PPD is characterized by depressed mood, loss of interest, sleep disturbances, psychomotor agitation or retardation, feelings of worthlessness, and even suicidal thoughts and behaviors in severe cases.^[5]^ Given the high prevalence and negative impact of PPD, effective treatment is clearly important.

Treatment of PPD includes pharmacotherapy, psychotherapy, or both, which is consistent with the treatment recommended in guidelines for major depression.^[6]^ However, these treatments vary in efficacy,^[7–9]^ are costly,^[10]^ and adverse events are common.^[11,12]^ Therefore, more effective and safer treatments for PPD are still needed. Alternative treatment strategies for PPD are receiving increasing attention, and acupuncture, in particular, has gained acceptance due to its effectiveness and safety.^[13]^

A number of systematic reviews (SRs)/meta-analyses (MAs) have reported on the efficacy of acupuncture for PPD, but their findings are inconsistent and the credibility of the evidence is unclear. SR/MA is considered the source of the highest level of evidence. However, not all SRs/MAs can provide reliable evidence, and low quality SRs/MAs may instead mislead decision makers.^[14]^ There is a gap between the use of evidence and its practical implementation in real-world dynamics. In addition, to ensure the standardization of evidence sources, measurement tools such as assessment of multiple SRs-2,^[15]^ preferred reporting items for systematic reviews and meta-analyses,^[16]^ and grading of recommendations, assessment, development, and evaluation system^[17]^ were released in 2007, 2009, and 2004, respectively. Where multiple SRs/MAs SRs/MAs are published for overlapping topics in a relatively short time frame, an overview is needed to systematically collate, evaluate, and synthesize the evidence from these SRs/MAs. Therefore, we will provide an overview of SRs/MAs on acupuncture for PPD.

## 2. Materials and Methods

### 2.1. Patient and public involvement

Patients and public will not be involved.

### 2.2. Protocol and registration

The method used for this protocol will follow the Cochrane Handbook.^[18]^ This protocol has been registered on the PROSPERO (CRD42021291289).

### 2.3. Criteria for including reviews

#### 2.3.1. Type of studies.

SRs/MAs enrolled random control trails investigating the therapeutic effects of acupuncture for PPD. No language constraints are placed on this study.

#### 2.3.2. Types of participants.

Participants are diagnosed with PPD by any recognized guideline, regardless of age, course of the disease, sex, or race.

#### 2.3.3. Types of interventions.

Intervention of interest is the use of acupuncture in PPD, acupuncture group could be with or without conventional medication, with conventional medication as the control.

#### 2.3.4. Types of outcomes.

The outcome measures are the following: the Hamilton Rating Scale for Depression, Edinburgh Postnatal Depression Scale, effective rate, estradiol levels

### 2.4. Search strategy

We will search Medicine, Web of science, Cochrane Library, Embase, China National Knowledge Infrastructure, SinoMed, VIP, and Wanfang. References to the SRs under this topic will also be screened to track relevant studies. The medical subject headings and keywords related to acupuncture, PPD, and meta-analysis comprise the search strategy. Search strategies for each database can be found in Supplementary A.

### 2.5. Eligibility assessment and data extraction

**Figure 1.**
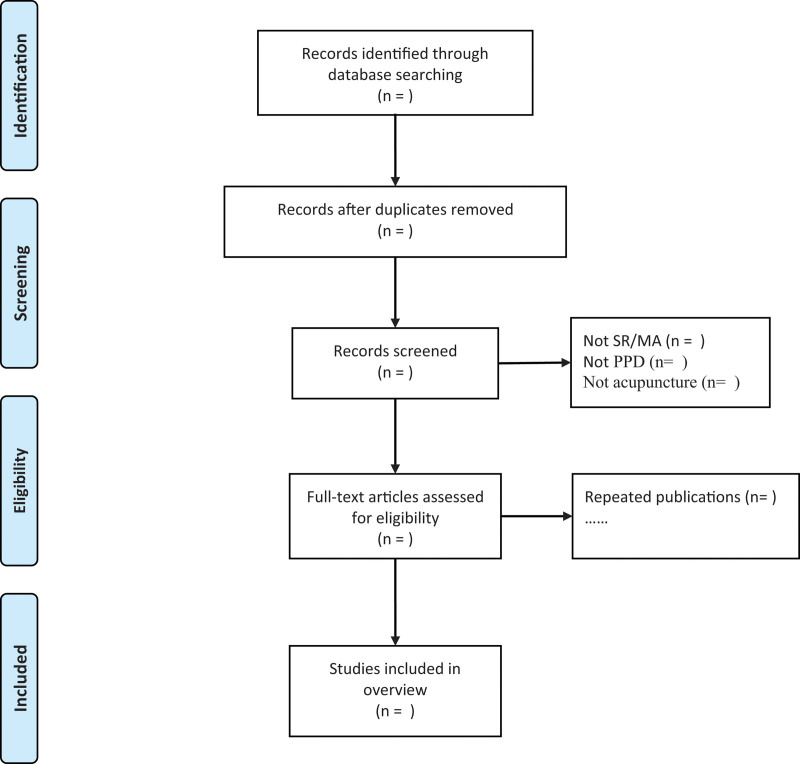
PRISMA flow chart for literature screening. MA = meta-analyses, PPD = postpartum depression, PRISMA = preferred reporting items for systematic reviews and meta-analyses, SR = systematic review.

The search results will be entered into NoteExpress to identify and remove duplicates. Literature screening will be performed by 2 independent reviewers (Fig. 1). Any disagreement will be adjudicated by an experienced third reviewer.

Data of the included studies will be extracted by 2 independent reviewers. The first author, year of publication, country, enrolled trails, tools for quality evaluation, interventions, comparisons, data synthesis methods, outcome measures, and main results will be extracted. Any disagreement will be adjudicated by an experienced third reviewer.

### 2.6. Quality assessment

The assessment of multiple SRs-2 tool^[15]^ will be applied to assess the methodological quality of the included studies by 2 independent reviewers. Any disagreement will be adjudicated by an experienced third reviewer. Furthermore, the reporting quality of the included SRs/MAs will be assessed using preferred reporting items for systematic reviews and meta-analyses checklists.^[16]^ Finally, the evidence quality will be assessed using the grading of recommendations, assessment, development, and evaluation system.^[17]^

### 2.7. Data synthesis

A descriptive analysis will be carried out in an overview.^[19]^ In SRs/MAs included in the overview, data from individual trials may have be pooled multiple times, therefore, meta-analysis will not be conducted in this study, but a narrative synthesis of the findings from the included reviews will be presented. For dichotomous data, findings from included reviews will be shown as risk ratio or odds ratio; for continuous data, as weighted mean difference or standard mean difference with 95% confidence intervals. The results of the quality assessment will be shown in tabular and graphical form.

## 3. Discussion

In China, acupuncture has been widely used for the clinical treatment of PPD. However, the researchers emphasize that this therapy is still not fully implemented in a real-world context. There is a gap between evidence-based clinical implementation of acupuncture and its actual implementation in real-world dynamics.

SR/MA are considered the gold standard for assessing the efficacy of clinical interventions, but the evidence derived from them is currently facing challenges due to the various risks of bias generated during the formation of evidence by SRs/MAs.^[14]^ High quality SRs/MAs can provide reliable evidence, while low quality SRs/MAs may instead mislead decision makers.^[20]^ Thus, Evidence of uneven quality leads to a gap between its use and its practical implementation in real-world dynamics. In recent years, a growing body of SRs/MAs based on randomized clinical trials has begun to analyze the evidence of acupuncture in the treatment of PPD. However, their results are not completely consistent, and their quality is uneven. These issues all compromise the development of clinical protocols and health decisions. Considering this status quo, an overview of SR/MA of this field is needed. The ultimate goal of this review is to provide a comprehensive evaluation of current evidence on multiple identical topics, to provide more focused high-quality evidence to evidence users, and to identify key flaws in evidence use.^[21]^ This protocol describes the detailed study design for performing an overview of SRs/MAs on acupuncture for PPD. Publication of this protocol will ensure reproducibility of the overview and benefit from feedback from peer expert review.

## Author contributions

Conceptualization: Fan Bu, Yonghou Zhao

Methodology: Jianbo Chai, Bing Bai

Supervision: Wanyu Wang

Validation: Jianbo Chai, Bing Bai

Writing - original draft: Fan Bu

Writing - review & editing: Yonghou Zhao
